# Graphene-Based Composites with Silver Nanowires for Electronic Applications

**DOI:** 10.3390/nano12193443

**Published:** 2022-10-01

**Authors:** Dimitra Giasafaki, Christina Mitzithra, Vassiliki Belessi, Theodora Filippakopoulou, Apostolos Koutsioukis, Vasilios Georgakilas, Georgia Charalambopoulou, Theodore Steriotis

**Affiliations:** 1 National Centre for Scientific Research “Demokritos”, 15341 Agia Paraskevi, Greece; 2 Department of Graphic Design and Visual Communication, Graphic Arts Technology Study Direction, University of West Attica, 12243 Egaleo, Greece; 3 Laboratory of Electronic Devices and Materials, Department of Electrical and Electronic Engineering, University of West Attica, 12244 Egaleo, Greece; 4 Department of Materials Science, University of Patras, 26504 Rio, Greece

**Keywords:** functionalized reduced graphene oxide, few-layered graphene, functionalized multiwalled carbon nanotubes, silver nanowires, conductive inks, gravure, printed electronics

## Abstract

Graphene/metal nanocomposites have shown a strong potential for use in electronic applications. In particular, the combination of silver nanowires (AgNWs) with graphene derivatives leads to the formation of an efficient conductive network, thus improving the electrical properties of a composite. This work focused on developing highly conductive hydrophilic hybrids of simultaneously functionalized and reduced graphene oxide (*f*-rGO) and AgNWs in different weight ratios by following two different synthetic routes: (a) the physical mixture of *f*-rGO and AgNWs, and (b) the in situ reduction of GO in the presence of AgNWs. In addition, the role of AgNWs in improving the electrical properties of graphene derivatives was further examined by mixing AgNWs with a hybrid of few-layered graphene with functionalized multiwalled carbon nanotubes (FLG/MWNT-*f*-OH). The studied materials showed a remarkable improvement in the overall electrical conductivity due to the synergistic effect of their components, which was proportional to the percentage of Ag and dependent on the procedure of the hybrid formation. One of the *f*-rGO/AgNWs composites was also selected for the preparation of gravure printing inks that were tested to determine their rheological and printing properties. All of the *f*-rGO/AgNWs composites were shown to be very promising materials for use as conductive inks for flexible electronics.

## 1. Introduction

Flexible printed electronics (FPEs) are expected to dominate soon in the field of electronics because they have shown great potential in a wide range of applications [1] including sensors and microheaters [2,3], transistors [4], light-emitting diodes (LEDs) and organic LEDs (OLEDs) [5,6], solar cells [7], and wearable electronics and e-textiles [8,9]. FPE development requires the connection of flexible conductive structures, interfaces, and contacts, which can be created through the printing of conductive, semiconductive, and dielectric nanomaterials on suitable substrates. Efforts to improve this technology have focused on the use of high-performance printing techniques such as inkjet, gravure, flexographic, and screen printing, as well as the formulation of highly conductive printable inks [10]. The required characteristics [11,12] of conductive inks, in addition to high conductivity, are flexibility and stretchability, good printability, compatibility with a wide range of substrates, long-term stability, a low cost, and the ability to be water-based in order to provide more environmentally friendly solutions. In this context, many types of conductive inks have been developed using different conductive carriers [13] such as metal nanoparticles (e.g., Au, Ag, and Cu), carbon nanomaterials including graphene and carbon nanotubes (CNTs), or polymers [12,14,15,16,17,18,19].

Metals stand out among these conductive nanomaterials, especially silver, which exhibits good oxidation resistance and excellent electrical (the highest of all metals at room temperature) and thermal conductivity. Due to these special characteristics, Ag nanostructures such as silver nanoparticles (AgNPs) and silver nanowires (AgNWs) are the most widely studied metal nanostructures for electronic applications [20,21]. In particular, nanoscale silver is widely used in conductive inks [22] that find use in the development of high-precision electronic circuits in several flexible substrates (e.g., smart labels, radio frequency (RFID) detectors, OLEDs, etc.) [20].

Compared to the silver nanoparticles used in traditional silver inks, the one-dimensional (1D) form of silver (i.e., AgNWs) could offer better performance in a variety of applications such as flexible and stretchable printable electronics, in which electrical conductivity, interconnections, contacts, and flexibility are essential. In general, 1D metal nanomaterials with a high aspect ratio have attracted great interest in both commercial and research fields due to their unique properties (e.g., electrical, thermal, optical, and magnetic), so they are therefore considered very promising candidates to be used in the areas of nanoelectronics, optoelectronics, nanophotonics, and micromechanics [23]. In particular, silver nanowires (AgNWs) represent a special combination of properties that make them suitable components in a vast range of applications including wearable electronics, smart sensors, energy harvesting devices, and optoelectronic displays [23,24]. Among the large variety of methods that have been reported for the preparation of AgNWs (such as hard and soft templating protocols, microwave-assisted techniques, ultraviolet irradiation, and hydrothermal synthetic routes), the polyol method is the most widely used because it is relatively low-cost, environmentally friendly, and simple [21,23,25,26,27].

However, apart from the major disadvantage of a high cost, silver-based conductive inks require high sintering temperatures and thus their use on temperature sensitive substrates such as paper and polyethylene terephthalate (PET) remains a challenge [20]. On the other hand, conductive carbon-based nanomaterials such as graphene and graphene derivatives are also promising materials for printed electronics due to their interesting properties such as high mechanical durability and flexibility, high inherent current mobility, and chemical corrosion resistance [3,21].

In this context, graphene has attracted great interest due to its high potential for electronic and optoelectronic applications. However, limitations arise due to its stability and extremely hydrophobic nature, making pure graphene inappropriate for water-based inks or requiring large amounts of surfactants for efficient dispersion. On the other hand, graphene oxide (GO) has numerous oxygenated groups that make it extremely hydrophilic and enable further chemical modification and functionalization. Although these oxygen functional groups are advantageous for some applications over pure graphene, for others they may be harmful because they impart a low electrical conductivity to the material. Various reduction routes have been followed to obtain reduced graphene oxide (rGO) by eliminating the oxygen groups in order to achieve similar electrical, thermal, and mechanical properties to those of pristine graphene [28]. A promising way to restore an extensive sp^2^ carbon network with good electrical properties while maintaining the high hydrophilicity of rGO, thus allowing its use in water-based conductive inks, could be the appropriate functionalization after or (even better) during the reduction process [2,16].

On this basis, novel carbon–metal heterostructures such as two-dimensional (2D) graphenic sheets with suitable surface functionalities and long 1D silver nanowires could be considered as a good combination. Such structures may in principle form a water-soluble, continuous conductive network with bridged gaps and enhanced electrical conductivity and flexibility [29].

Herein, we focused our efforts on developing a new generation of hybrid materials that may alleviate the technological limitations of existing gravure printing inks by combining multiple advantages that include: water dispersibility without the need for surfactants, a low sheet resistance without the necessity of sintering or annealing, easy and eco-friendly production, scalability, and cost efficiency [9,30,31,32,33,34,35]. In brief, we present the incorporation of flexible silver nanowires prepared using a polyvinylpyrrolidone (PVP)-mediated polyol method in the presence of halides onto functionalized reduced (by the sulfonated aromatic diamine, diamino-benzenesulfonic acid, DBSA) GO nanosheets (denoted as *f*-rGO_DBSA_), forming innovative hybrid materials with a high conductivity and hydrophilicity. Two different synthetic techniques were followed: (i) the physical mixture of the premade *f*-rGO_DBSA_ and the AgNWs; and (ii) the in situ reduction of GO in the presence of AgNWs. Different metal loadings were applied in order to study the margins for increasing the conductivity of the new materials. In addition, AgNWs were mixed with a conductive few-layered graphene (FLG) hybrid with functionalized multiwalled carbon nanotubes (FLG/MWNT-*f*-OH) in several ratios. This hybrid, which was formed by doping FLGs prepared via liquid exfoliation of graphite with catechol-functionalized MWNTs, was previously reported as a hydrophilic, conductive graphene hybrid [17]. All nanocomposites were fully characterized using various techniques while their electrical performances were also evaluated. In addition, large-scale production of a selected system was attempted for the preparation of a low-cost water-based conductive ink, for which the sintering process would not be necessary.

## 2. Materials and Methods

### 2.1. Materials

Graphite (powder, synthetic, particle size < 20 μm), polyvinylpyrrolidone, 2,4-diamino-benzenesulfonic acid (2,4-DBSA) (≥97.0%), and dimethylformamide (DMF) were acquired from Sigma-Aldrich (St. Louis, MO, USA). Sulfuric acid (95–97%), potassium chlorate (>99.0%), silver nitrate, sodium chloride, and sodium bromide were supplied by Merck (Darmstadt, Germany). Nitric acid (65%) was purchased from Riedel-de Haën (Munich, Germany) and ethylene glycol from Penta (Prague, Czech Republic). All reagents were of analytical grade and no further purification was required. The water-based resin emulsions used in this work are a product under development that contains mainly a mixture of styrene–acrylate and acrylic resins (called R-UDP). The solid content of the R-UDP was measured at appr. 42%.

### 2.2. Synthesis of Graphite Oxide

For the synthesis of GO, a modified Staudenmaier method [36] was followed. In particular, 2 g of graphite microparticles was added to a mixture consisting of 40 mL of nitric acid (65%) and 80 mL of sulfuric acid (95–97%). The reaction took place in a Pyrex beaker immersed in an ice bath under vigorous stirring; 40 g of potassium chlorate was then slowly added while maintaining the cooling conditions. After completion of the oxidation (~18 h), the mixture was washed with deionized (DI) water, centrifuged at 9000 rpm, and dried at room temperature to obtain the final product.

### 2.3. Simultaneous Reduction and Chemical Functionalization of GO (f-rGO_DBSA_)

#### 2.3.1. *f*-rGO_DBSA_ Synthesis

The *f*-rGO_DBSA_ material was prepared as reported by Belessi et al. [16]. More specifically, 75 mg of GO was dispersed in DI water (1 mg/mL) and stirred for 24 h; afterward, the dispersion was also ultrasonicated (Branson 3800, Brookfield, USA, 110 W, 40 kHz) for 20 min to achieve further exfoliation. Subsequently, 2,4-diaminobenzenesulfonic acid (DBSA) in a mass ratio of DBSA:GO = 3:1 was added to the suspension and the mixture was refluxed for 2 h under magnetic stirring. The reaction mixture was cooled to room temperature (RT) and centrifuged at 9000 rpm. The obtained solid was washed thoroughly with DI water, ethanol, and acetone and vacuum-filtered using 0.45 μm nylon membrane filters (Fiorini). The final product was kept as an aqueous dispersion for further use.

#### 2.3.2. *f*-rGO_DBSA_ Scale-Up Process

The procedure described above was also followed in order to produce *f*-rGO_DBSA_ on a large scale but starting with 0.5 g of GO. In brief, DBSA was added in a mass ratio of DBSA:GO = 3:1 in a 1 mg/mL aqueous dispersion of the exfoliated GO and then the mixture was refluxed under magnetic stirring for 2 h. The large batch obtained after washing, filtration, and redispersion in DI water was used to prepare the *f*-rGO_DBSA_/AgNWs nanocomposites using the physical mixture method.

### 2.4. Synthesis of Silver Nanowires

The AgNWs were developed according to the process reported by Cao et al. [37]. In brief, 5.2 mmol of polyvinylpyrrolidone (PVP) was dissolved in 19 mL of ethylene glycol (EG) under magnetic stirring. Subsequently, 0.6 mL and 1.6 mL of two premade solutions of 0.01 M NaCl and 0.005 M NaBr in EG, respectively, as well as 3.5 mmol of AgNO_3_, were added. The reaction mixture was refluxed for approximately 18 min using an oil bath at 175 °C while stirring. The final product was collected after centrifugation and washing with ethanol at 9000 rpm for 5 min and resuspended in ethanol for further use.

### 2.5. Synthesis of f-rGO_DBSA_/AgNWs Nanocomposites

Two different approaches were followed for preparing the *f*-rGO_DBSA_/AgNWs hybrid materials with three different metal loadings (10, 30, and 50 wt %) as described below.

#### 2.5.1. Physical Mixture Method

In this case, the required amount of the preformed AgNWs suspension was mixed with the premade *f*-rGO_DBSA_ dispersion and the mixture was stirred for 24 h. The resulting samples are designated in the text as “*f*-rGO_DBSA_/AgNWsX% phys-mix”, where “X” stands for 10, 30, or 50 corresponding to the metal loading (10, 30, or 50 wt %, respectively) and “phys-mix” means the “physical mixture” synthetic method.

#### 2.5.2. In Situ Method

In this case, an appropriate quantity of the AgNWs suspension was added to a mixture consisting of a 1 mg/mL aqueous dispersion of the exfoliated GO and the DBSA in a mass ratio of GO:DBSA = 1:3, as used for the preparation of *f*-rGO_DBSA_. The reaction was then refluxed for 2 h under stirring; the solids obtained after cooling to RT by centrifugation at 9000 rpm were vacuum-filtered and washed with DI water, ethanol, and acetone. The resulting hybrids are denoted in the text as “*f*-rGO_DBSA_/AgNWsX% in situ”, where “X” stands for 10, 30, or 50 corresponding to the silver loading (10, 30, or 50 wt %, respectively) and “in situ” means synthesis method.

#### 2.5.3. Scale-Up of *f*-rGO_DBSA_/AgNWs10% In Situ Nanocomposite

The *f*-rGO_DBSA_/AgNWs10% in situ hybrid was chosen to be produced on a larger scale. For this reason, a procedure similar to the one described above was used. Specifically, the required amount of the AgNWs suspension (to achieve 10 wt % of metal loading on the hybrid system) was added to a 4 mg/mL aqueous dispersion of 2 g of the exfoliated GO containing DBSA in a mass ratio of DBSA:GO = 3:1; the mixture was then refluxed under magnetic stirring for 2 h. The large batch obtained after centrifugation, washing, filtration, and redispersion in DI water was used to prepare a water-based conductive ink with the *f*-rGO_DBSA_/AgNWs10% in situ nanocomposite.

### 2.6. Synthesis of FLG/MWNT-f-OH/AgNWs Nanohybrids

#### 2.6.1. Synthesis of Hybrid FLG/MWNT-*f*-OH

The FLG was prepared via exfoliation of graphite in DMF with bath sonication for 1 h. The FLG material was isolated from the supernatant after one hour for the settlement of the unexfoliated graphite. The hybrid FLG/MWNT-*f*-OH was prepared by mixing FLG and MWNT-*f*-OH dispersed in dimethylformamide in a ratio of 8:2. The mixture was stirred overnight and the hybrid was isolated via centrifugation (10,000 rpm) and redispersed in water. The functionalization of the MWNTs was described elsewhere [17].

#### 2.6.2. Physical Mixture of AgNWs with FLG/MWNT-*f*-OH

The FLG/MWNT-*f*-OH/AgNWs nanohybrids were prepared by mixing a water dispersion of the graphene hybrid FLG/MWNT-*f*-OH with AgNWs. The mixture was homogenized via vortexing for 24 h. Three samples were prepared with AgNWs percentages of 10, 30, and 50 wt %.

### 2.7. Preparation of f-rGO_DBSA_/AgNWs Ink Formulations with Different Resin Content

The scaled-up *f*-rGO_DBSA_/AgNWs10% hybrid was used for preparing two conductive ink formulations by mixing an appropriate amount of the hybrid with resin emulsions and water while considering that it was desirable to keep the ratio of conductive/nonconductive components as high as possible. Specifically, 0.97 g (2.3 g) of the resin, 1.0 g (1.0 g) of the hybrid, and 10.4 g (11.4 g) of water were mixed to prepare two inks with a ratio of pigment solids/resin solids of 70/30 and 50/50, respectively. The two inks were respectively called Ink_P70/R30_ and Ink_P50/R50_. The ink formulations were prepared by gradually adding pigment to the mixture of water and resins using a homogenizer (Witeg Labortechnik GmbH, Wertheim, Germany, up to 27,000 rpm) and a vortexer (LLG-uniTEXER, Lab Logistics Group GmbH, Meckenheim, Germany, with universal attachment, 3000 rpm) as the laboratory mixing equipment.

### 2.8. Characterization

A D-500 Siemens (Munich, Germany) diffractometer (CuK_α_, λ = 1.54 Å) was used for the structural characterization of the samples by employing the X-ray diffraction (XRD) technique. A JEOL JSM-70401F (JEOL Ltd, Tokyo, Japan) system equipped with the Gentle Beam mode was employed to study the surface morphology of the materials using the electron scanning microscopy (SEM) technique. An Xplore-15 SDD detector (Oxford Instruments) with a surface of 15 mm^2^ at an acceleration voltage of 20 kV was used to investigate the elemental composition of the hybrids using energy dispersive spectroscopy (EDS). A FEI Talos F200i S/TEM field emission gun scanning electron microscope (Thermo Fisher Scientific, Waltham, MA, USA) operating at 200 kV and lacey carbon films on 200 mesh copper grids were utilized to obtain the transmission electron microscopy (TEM) images. A SETARAM SETSYS Evolution 18 Analyzer (Setaram Instrumentation, Caluire-et-Cuire, France) was used to study the thermal behavior and determine the metal concentration of the samples via a thermogravimetric analysis (TGA); the measurements were carried out in a temperature range of 25–1100 °C with a heating rate of 10 °C/min under an air flow (16 mL/min) using Al_2_O_3_ crucibles.

### 2.9. Inks Rheological Characterization

A rotational rheometer (Malvern Kinexus Pro+, Malvern Panalytical Ltd., Worcestershire, UK) was used for the rheological characterization of the prepared inks. A cone (40 mm diameter and 4° angle) and plate geometry was used. Shear-rate ramp tests were carried out; the range of the shear rate was kept between 0.01 and 1000 s^−1^ with an interval of 10 points per decade; i.e., 50 points for each flow curve. The temperature was kept constant at 25 °C.

### 2.10. Gravure Printing

Gravure printing tests were performed by using the IGT G1-5 printability tester (IGT Testing Systems, Almere, Netherlands) with a chromium-plated printing cylinder named by IGT as 402.226 (60, 80, 100, and 140 lines cm^−1^; screen angle 53; stylus angle 130; and cell volumes of 16, 11, 9, and 7 mL m^−2^). The printing force between the printing disc and the substrate was 200 N and the printing speed was 0.6 m/s. The substrate was C 2846 coated paper, which is widely used as a standard paper in the printing industry; this was obtained from IGT Testing Systems (150 g/m^2^).

### 2.11. Electrical Measurements

A Loresta-GX MCP-T700 (Nittoseiko Analytech Co., Ltd., Kanagawa, Japan) resistivity meter was used to measure the sheet resistance (R_s_) of all samples via the 4-point probe method after deposition of 400 μg of each sample on coated paper, drying at room temperature (RT), and “polishing” using mild pressure so that films with an average thickness of about 17μm were formed. The R_s_ values of the printed substrates were measured using a 4-point probe system (Lucas Labs Pro4 Resistivity System, Lucas Signatone Corp., Gilroy, CA, USA) and a Keithley 2400 Source Meter (Keithley Instruments, Cleveland, OH, USA).

## 3. Results and Discussion

### 3.1. X-ray Diffraction Measurements

**Figure 1:**
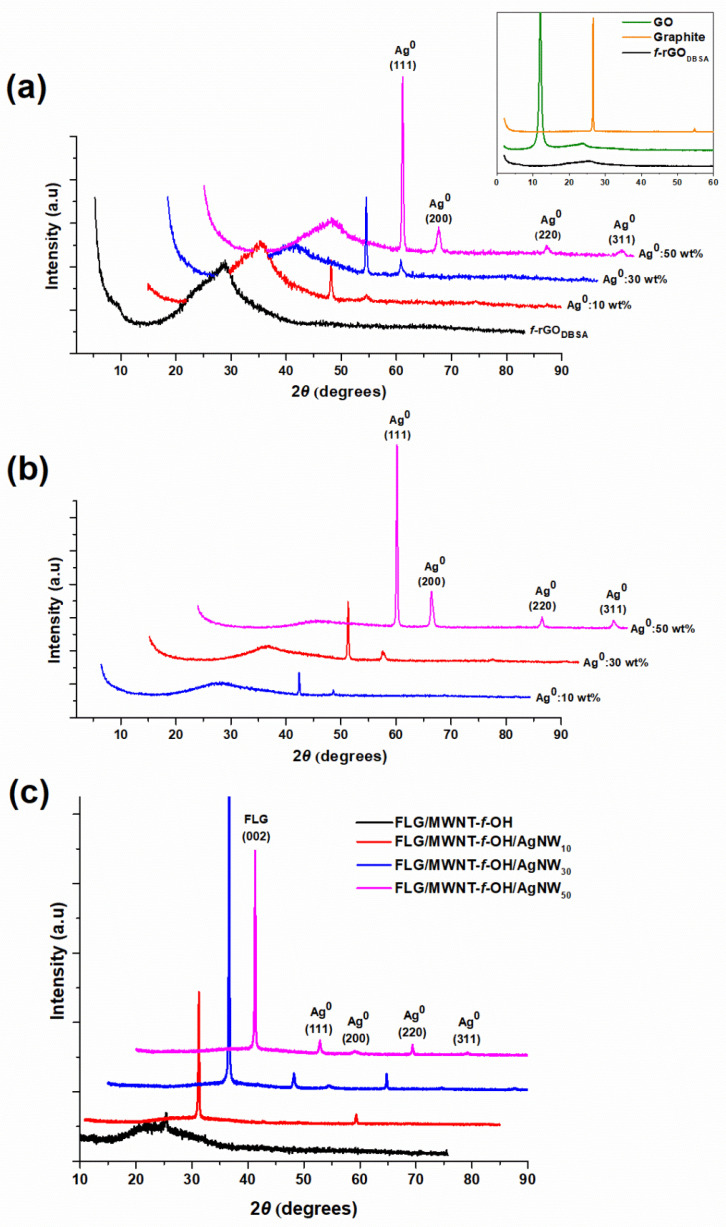
XRD patterns of *f*-rGO_DBSA_/AgNWs hybrid systems with three different metal loadings (10, 30, and 50 wt %) prepared with (**a**) the physical mixture method and (**b**) the in situ reduction method. XRD patterns of graphite, GO, and *f*-rGO_DBSA_ are also given for comparison reasons (inset of (**a**)); (**c**) XRD patterns for FLG/MWNT-*f*-OH/AgNWs nanohybrids with different metal loadings (0, 10, 30, and 50 wt %).

Figure 1a,b depicts the XRD patterns for the *f*-rGO_DBSA_/AgNWs hybrid materials prepared using the two different synthetic approaches (physical mixture and in situ reduction) with three different silver loadings (10, 30, and 50 wt %). The four peaks centered at about 38.1°, 44.4°, 64.4°, and 77.4° corresponding to the (111), (200), (220), and (311) lattice planes of the face-centered cubic (fcc) Ag (JCPDS 04-0738) [38] verified the presence of metallic silver on the surface of the *f*-rGO_DBSA_ sheets, thus confirming the successful preparation of the *f*-rGO_DBSA_-Ag nanocomposites.

Moreover, as shown in the XRD patterns in the Figure 1a inset, pure graphite exhibited a distinct diffraction peak at ca. 26.6° and a smaller peak at approx. 54.8° corresponding to the (002) and (004) planes, respectively [39]. After treatment with the modified Staudenmaier method, the XRD pattern for GO revealed a strong diffraction peak at ca. 11.98° corresponding to an interlayer distance of 0.738 nm [16] and a weak XRD peak at about 23.8° with an interplanar spacing of 0.373 nm. The increment in the layer distance indicated that graphite had been successfully exfoliated and converted into GO with a large amount of oxygen groups on its surface [16,38,39]. After reduction of GO with DBSA, the prominent peak at around 12° vanished and the *f*-rGO exhibited a broad peak at ca. 25.6° corresponding to the (002) plane of graphite with an interlayer spacing of 0.348 nm, indicating the removal of some oxygen functional groups and a slight degree of restacking by the conversion of GO to *f*-rGO_DBSA_ [16,40].

In the case of the *f*-rGO_DBSA_/AgNWs hybrids, a broad diffraction peak for *f*-rGO was also present and slightly shifted to smaller angles (24–25° for physical mixture and 23–24° for in situ composites). The average interplanar spacing of the graphene sheets was increased in both cases (to 0.363 nm for the physical mixture method and 0.378 nm for the in situ approach), in accordance with the literature data [41].

Finally, the XRD patterns for the FLG/MWNT-*f*-OH/AgNWs nanohybrids (Figure 1c) contained three of the characteristic peaks for AgNWs and a peak at 26.4°, which was attributed to the partly graphitic character of the FLGs [42,43,44].

### 3.2. SEM/TEM Analysis

**Figure 2:**
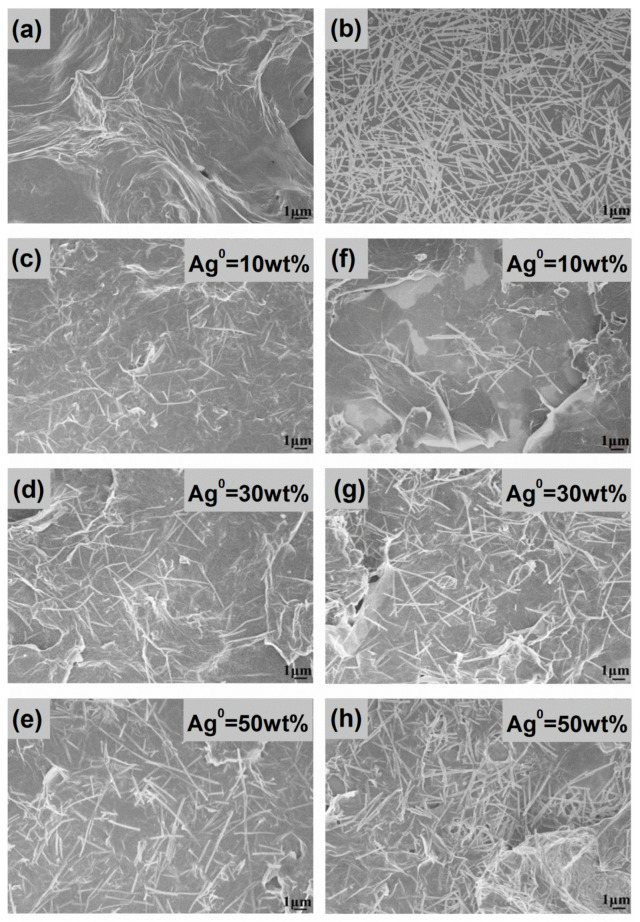
SEM images of (**a**) pristine *f*-rGO_DBSA_; (**b**) preformed AgNWs; (**c**–**e**) hybrid materials of *f*-rGO_DBSA_/AgNWs physical mixture; and (**f**–**h**) *f*-rGO_DBSA_/AgNWs in situ, with silver loadings of 10, 30, and 50 wt %, respectively.

Initially, the surface morphologies of both the *f*-rGO_DBSA_ and the preformed AgNWs were investigated via SEM analysis. The SEM micrograph for *f*-rGO_DBSA_ (Figure 2a) revealed the typical wrinkled-like morphology of thin nanosheets, with some agglomerated layers caused by the restacking process after reduction. Figure 2b demonstrates the morphology of the silver nanowires that were utilized to synthesize the nanocomposites. We observed that the length of the as-produced AgNWs ranged from 3 to 20 μm and the diameter ranged from 80 to 150 nm. These results, combined with the relatively handy and fast polyol method we followed, indicated that the synthesis of the AgNWs was successfully achieved in a short time and in an easy way, presenting a satisfactory aspect ratio.

The SEM images of the hybrid systems developed by both the physical mixture (Figure 2c–e) and in situ (Figure 2f–h) methods showed that AgNWs were successfully anchored on the surface of graphene sheets; some of them were either inserted between the sheets or pierced them, favoring the formation of a random interconnected conductive network that provided intrinsic electric characteristics to the materials.

**Figure 3:**
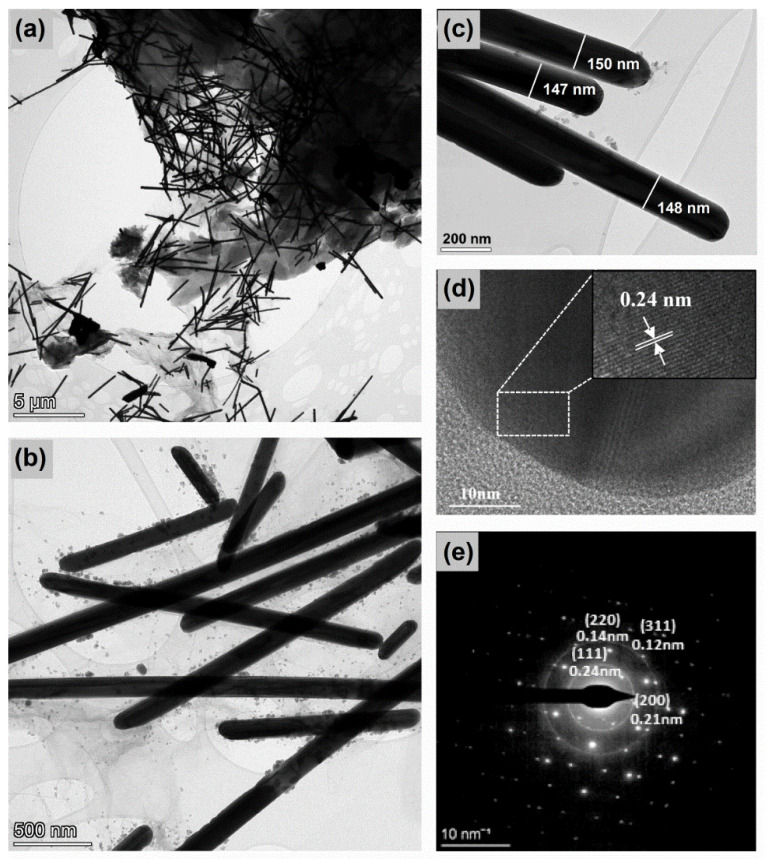
(**a**–**c**) Representative TEM images of *f*-rGO_DBSA_/AgNWs50% system prepared via the in situ method; (**d**) HRTEM of the tip region of a single silver nanowire; (**e**) typical SAED pattern of the tip region.

A further investigation of the morphology and crystal structure of the nanocomposites was conducted via a TEM analysis, as shown in Figure 3. It was clearly presented that the transparent wrinkled *f*-rGO sheets were successfully decorated with abundant flexible AgNWs anchored onto the surface of the *f*-rGO layers and residing between them (Figure 3a). The TEM images of the silver nanowires (Figure 3b,c) verified the length and diameter range that was estimated based on the SEM analysis. The presence of a small amount of silver nanoparticles was somewhat unavoidable because they were byproducts of the AgNWs synthetic process and their complete elimination during the purification procedure was quite difficult [24]. Figure 3c shows the detailed morphology of the plain silver nanowires, which presented a pentagonal end face that comprised five (111) planes [37] and a smooth surface, suggesting the complete removal of the thin PVP shell around them, which ensured the growth of one-dimensional AgNWs [41].

High-resolution TEM (HRTEM) images (Figure 3) revealed a lattice spacing of 0.24 nm corresponding to the (111) crystal plane of the fcc lattice of metallic silver. The selected area electron diffraction (SAED) image (Figure 3d) that was obtained by focusing the electron beam onto the tip region of a single AgNWs demonstrated diffraction rings with distinct bright spots, indicating the good crystallinity of the AgNWs. The diffraction spots corresponding to the (111), (200), (220), and (311) fcc lattice planes of the AgNWs were denoted in the SAED pattern.

**Figure 4:**
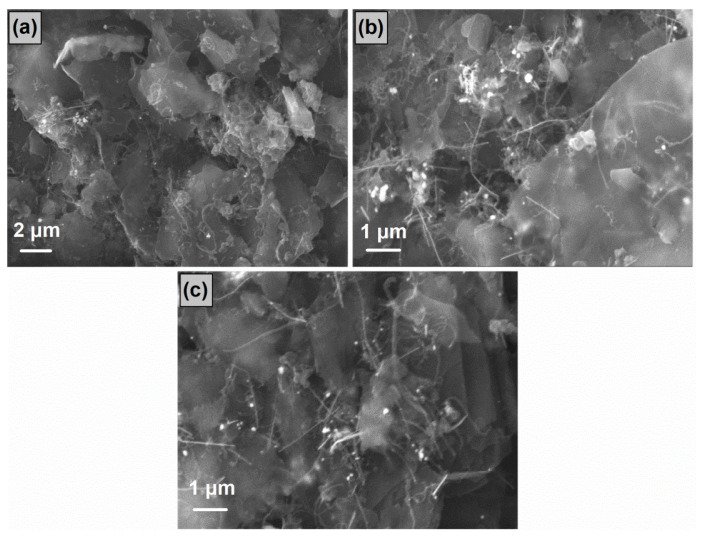
SEM images of FLG/MWNT-*f*-OH/AgNWs nanohybrids with (**a**) 10, (**b**) 30, and (**c**) 50 wt % AgNWs.

A similar image was observed in the SEM micrographs of the FLG/MWNT-*f*-OH/AgNWs nanohybrids (Figure 4). Depending on their percentage, the AgNWs were randomly deposited on the FLG nanosheets; however, they were barely discriminated from the very similarly shaped MWNT-*f*-OH.

Table 1 summarizes the average wt % concentration of Ag on the hybrid FLG/MWNT-*f*-OH/AgNWs samples as obtained from different regions.

### 3.3. Thermogravimetric Analysis

**Figure 5:**
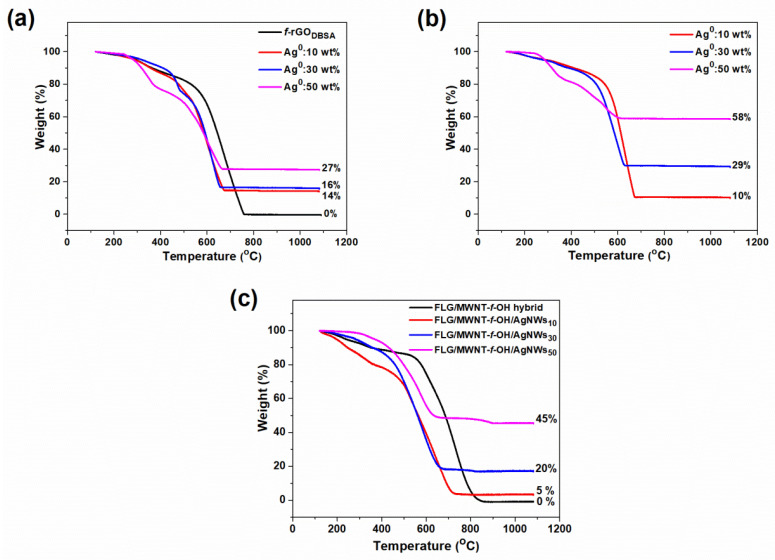
TGA curves for *f*-rGO_DBSA_/AgNWs hybrid systems with three different metal loadings (10, 30, and 50 wt %) prepared with the (**a**) physical mixture and (**b**) in situ method. The curve for the pristine *f*-rGO_DBSA_ is also given for comparison (Figure 5a). (**c**) TGA curves for FLG/MWNT-*f*-OH and FLG/MWNT-*f*-OH/AgNWs.

The metal loading of the composite materials was examined via thermogravimetric analysis (TGA) under air flow; the TGA curves on a dry sample basis (120 °C) are shown in Figure 5.

The initial small weight loss in both cases (physical mixture and in situ hybrids) in the temperature range of 120–300 °C could be attributed to the thermal decomposition of some oxygen-containing groups on the *f*-rGO nanosheets such as epoxy, hydroxyl, carboxyl, and carbonyl functionalities. The subsequent relatively greater weight loss up to ~400 °C could be associated with the removal of some more stable aryl sulfonic functional groups on the graphenic surface [45,46]. These results suggested that most of the oxygen groups on the GO surface were efficiently removed (in accordance with the XRD analysis) and at the same time new and more stable functional groups were anchored on the rGO surface, indicating that the reduction and simultaneous functionalization process by DBSA was successful. The following significant weight loss up to ca. 700 °C corresponded to the pyrolysis of the graphenic structure to CO and CO_2_. The final residual weight that remained constant after 800 °C was due to the presence of metallic silver on the *f*-rGO surface and provided information on the metal loading of the hybrid materials. The thermal gravimetric curves of both the *f*-rGO_DBSA_ and *f*-rGO_DBSA_/AgNWs displayed almost similar patterns. However, the increase in metal loading seemed to thermally destabilize the samples (showing lower burn-off temperatures in the initial steps with increasing silver content), probably due to some sort of catalytic effect. In most of the cases, the wt % of silver loading was found to be close to the theoretical one, especially for the hybrids prepared using the in situ approach. Nevertheless, in some cases, the estimated concentration appeared to have exceeded the amount of metal that was calculated to be used in the composition, while in other cases it was lower. Since the preformed AgNWs are maintained in the form of ethanol dispersion, an error in calculations is very likely, mainly due to possible agglomerates that occur over time [47].

The TG analysis of the FLG/MWNT-*f*-OH/AgNWs samples was characterized by the main weight loss between 400 and 700 °C, which was attributed to the pyrolysis of the carbon component. The remaining mass was associated with the amount of AgNWs in the samples. Regarding the Ag wt % loading, the results derived from the TGA and EDS analyses were fully consistent. Only in the case of the highest loaded sample was there a deviation that could be justified in view of the different nature of the two techniques (bulk vs. surface).

### 3.4. Water Dispersibility and Stability of f-rGO_DBSA_ and f-rGO_DBSA_/AgNWs Hybrids

**Figure 6:**
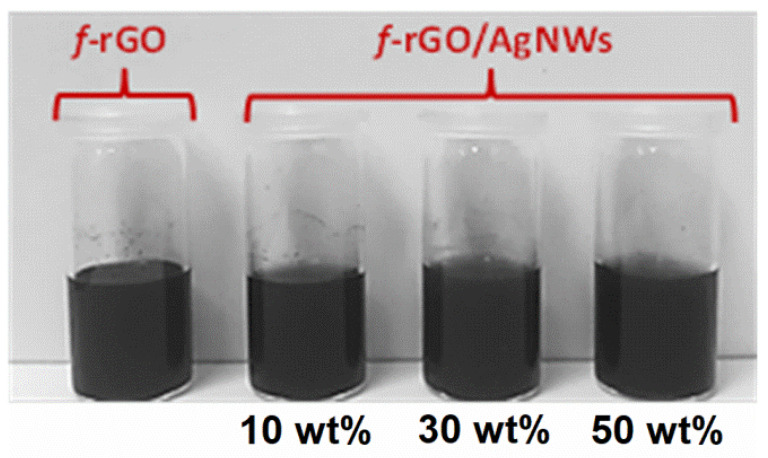
Indicative photo of *f*-rGO_DBSA_ and *f*-rGO_DBSA_/AgNWs hybrid systems with three different metal loadings (10, 30, and 50 wt %) dispersed in water (1 mg/mL) after three months.

Figure 6 illustrates the aqueous dispersion of the *f*-rGO_DBSA_ and the hybrid *f*-rGO_DBSA_/AgNWs systems with 10, 30, and 50 wt % metal content after 3 months. The *f*-rGO_DBSA_ and the *f*-rGO_DBSA_/AgNWs hybrids displayed a high water dispersibility and long-term stability, verifying that they could be used as pigments in water-based inks for environmentally friendly applications. The simultaneous reduction and surface functionalization of GO by diamino benzene sulfonic acid led to highly dispersible and stable *f*-rGO products [16]. This knowledge was successfully exploited in this work: all the hybrid materials prepared using the two different synthetic routes (both the physical mixture and the in situ methods) were endowed with a high dispersibility and stability.

### 3.5. Electrical Measurements 

**Figure 7:**
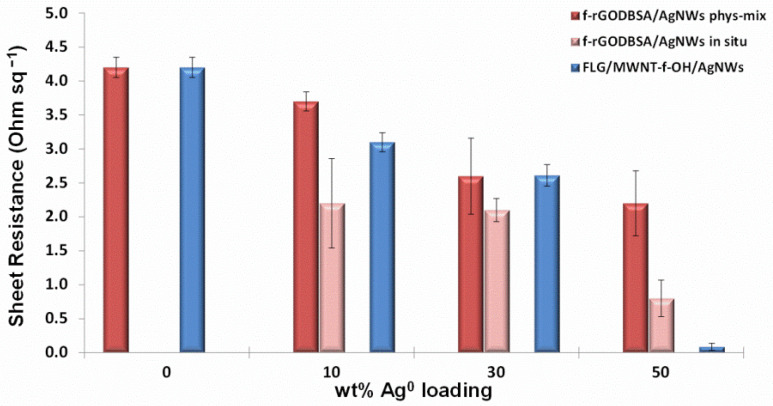
Sheet resistance of *f*-rGO_DBSA_, FLG/MWNT-*f*-OH, *f*-rGO_DBSA_/AgNWs, and FLG/MWNT-*f*-OH/AgNWs hybrid systems with three different metal loadings (10, 30, and 50 wt %).

The electrical performances of the as-prepared materials were evaluated via sheet resistance (R_s_) measurements using the four-probe method. The R_s_ was measured at least three times and the mean values were recorded. The results of the measurements are presented in Figure 7. It was previously reported [16] that chemical functionalization affects the electrical properties of graphene materials. Indeed, the reduction of GO with amino and aryl sulfonic groups (DBSA) led to the development of the *f*-rGO_DBSA_ system with a notably low sheet resistance (R_s_ = 4.2 ± 0.15 Ohm sq^−1^).

The enrichment of the *f*-rGO_DBSA_ with silver nanowires further reduced the sheet resistance even when the content of the metal was only 10 wt %. The lowest R_s_ was achieved in the case of the *f*-rGO_DBSA_/AgNWs50% in situ hybrid (R_s_ = 0.8 ± 0.27 Ohm sq^−1^), which was reduced almost by 80% compared to the corresponding R_s_ of the *f*-rGO_DBSA_. It was previously suggested [48] that 1D AgNWs can bridge the gap between 2D conductive rGO nanosheets, creating an effective conductive network (in both vertical and planar directions); consequently, more effective electron-transport paths can be obtained. However, this mechanism seemed to be better served in the case of the *f*-rGO_DBSA_/AgNWs in situ systems for which lower R_s_ values were recorded. We assumed that the presence of AgNWs in the reaction mixture during the reduction of GO led to their functionalization by DBSA by providing functional groups for AgNWs as well, thus creating conditions for grafting onto the *f*-rGO nanosheets [38,49] rather than simply being adsorbed on the surface, as in the case of the physical mixture method.

The sheet resistance of the FLG/MWNT-*f*-OH hybrid was also remarkably reduced after mixing with AgNWs.

Since the in situ *f*-rGO_DBSA_/AgNWs nanocomposite with 10 wt % metal loading exhibited a sufficiently low sheet resistance (R_s_ = 2.2 ± 0.66 Ohm sq^−1^), it was selected to be produced at a larger scale and was used as pigment for the development of the water-based conductive ink.

### 3.6. Characteristics of the Up-Scaled f-rGO_DBSA_/AgNWs10% Hybrid

**Figure 8:**
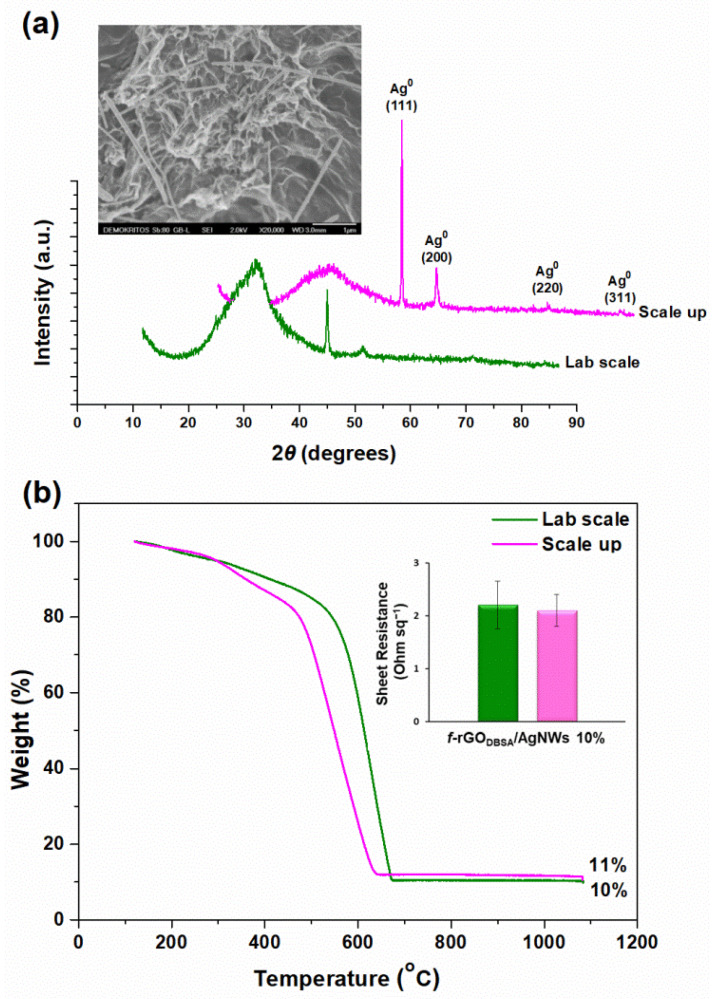
(**a**) XRD pattern of the up-scaled *f*-rGO_DBSA_/AgNWs10% hybrid in comparison with that of the lab-scaled hybrid (inset: SEM image of the scaled-up nanocomposite); (**b**) TGA curve for the up-scaled hybrid in comparison with that of the lab-scaled hybrid (inset: corresponding sheet resistance measurements).

The characterization of the up-scaled hybrid material used as pigment for the development of the water-based conductive ink is presented in Figure 8. We observed that the large batch retained the properties of the lab-scale nanohybrid according to its structural and morphological characteristics (Figure 8a), as well as based on the sheet resistance measurements (Figure 8b, inset). Finally, the TG analysis (Figure 8b) showed that the experimental Ag concentration was very close to the theoretical one (10 wt %) and thus the decoration of the nanocomposite with AgNWs was also successful during the scaled-up process.

### 3.7. Rheological Characteristics

**Figure 9:**
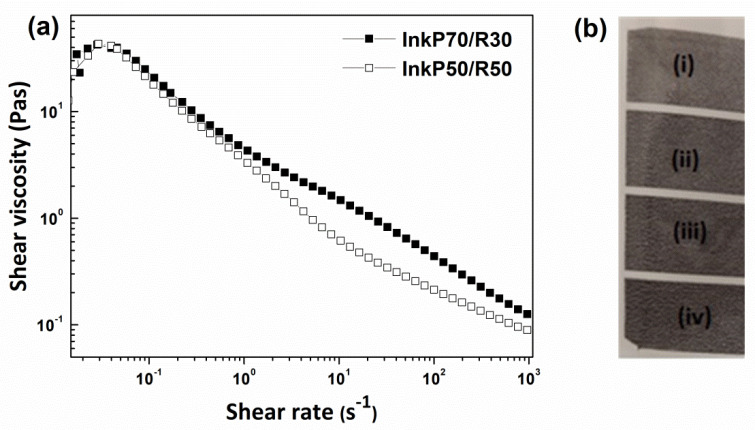
(**a**) Viscosity of both inks (Ink_P70/R30_ and Ink_P50/R50_) as a function of shear rate with the ratio of pigment solids/resin solids as a parameter; (**b**) gravure-printed item with four zones (i–iv) of different thicknesses of the ink layer (thickness increases from i to iv).

The rheological properties of an ink are important in defining how it will behave during storage and application (gravure printing). Shear-rate ramp tests were carried out using both conductive inks (Ink_P70/R30_ and Ink_P50/R50_) by monitoring the shear viscosity and shear stress as a function of the variable shear rate. The results are shown in Figure 9a, where the variation in viscosity versus the shear rate is presented for the two prepared inks.

The prepared inks, which had different pigment solids/resin solids ratios, exhibited shear-thinning properties; i.e., the viscosity decreased as the shear rate increased. For inks, shear thinning (pseudoplastic behavior) is desirable and occurs over a wide range of applied shear rates because such rates are very low under storage conditions but are high at the printing cylinder [50]. The viscosity of inks is required to be high during storage to ensure that settling does not occur, while it must be lower during application to ensure that a uniform film is applied [51,52]. Both inks that were developed in this work showed similar viscosities at a low shear rate, but as the shear rate increased, the viscosity curves diverged and the viscosity of the Ink_P70/R30_ showed higher values than the Ink_P50/R50_. This behavior was attributed to the higher pigment content (8.1%) of the Ink_P70/R30_ compared to the Ink_P50/R50_ (6.8%). It is known that as the solids content of a pigment dispersion increases, the viscosity values also increase [52,53].

### 3.8. Printing Results

Both hybrid conductive inks (Ink_P70/R30_ and Ink_P50/R50_) were printed using the gravure method. Figure 9b presents the gravure-printed item with four zones of different thicknesses of the ink layer (i–iv, with iv as the thickest). The R_s_ values obtained for the i, ii, iii, and iv lines were 16.3, 7.5, 3.5, and 8.0 kΩ sq^−1^ and 17.0, 9.0, 6.8, and 1.5 kΩ sq^−1^ when the Ink_P70/R30_ and Ink_P50/R50_ were applied, respectively. The R_s_ values of the printed zones increased as the thickness of the printed ink layers decreased. The high R_s_ value of 8.0 kΩ sq^−1^ derived from the line “iv” (when the Ink_P70/R30_ was used) is due to the poor printing quality. Generally, the printing quality was improved when the ratio of pigment solids/resin solids was decreased from 70/30 to 50/50 as the resin solids were simultaneously doubled from 3.48 to 6.92.

**Figure 10:**
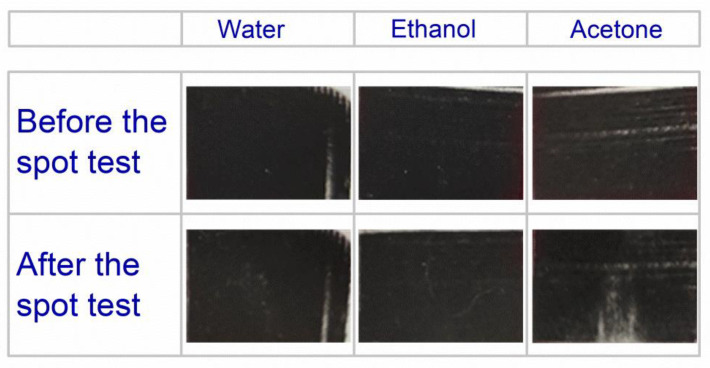
Stability test on printed specimens with different solvents.

The stability of the printed specimens on the paper substrate was tested according to the internal standards of the Druckfarben Hellas SA Printing Inks and Coating Manufacturing Company, which are based on ASTM D 1647 and ASTM D 2248 standards [54,55]. In brief, the test method was carried out by applying drops of water, ethanol, and acetone on the printed area and allowing them to remain for 10 min. Water and ethanol did not cause any visible damage or dissolution of the conductive ink while acetone caused a small one (Figure 10).

## 4. Conclusions

The design of suitable formulations of printable nanomaterial-based conductive inks has attracted great attention in both the commercial and research fields of flexible and stretchable electronics. Indeed, many metal- and carbon-based nanomaterials with a high conductivity such as metal nanoparticles, metal nanowires, graphene, and graphene derivatives have been proposed and used in printed electronic devices. In particular, hybrid systems of metal–carbon nanocomposites are considered to be a very promising combination. In this work, hybrid materials consisting of appropriately functionalized reduced graphene oxide (*f*-rGO_DBSA_) formed in a way to exhibit both a high conductivity and hydrophilicity were combined with flexible silver nanowires (AgNWs) in different concentrations by following two different synthetic approaches: the physical mixture and the in situ reduction method. All samples were fully characterized in terms of their morphological and structural features, while their sheet resistance (R_s_), water dispersibility, and long-term stability were also evaluated. 

The functionalization of the graphene sheets with amino and aryl sulfonic groups positively affected the electrical properties and water dispersibility of the rGO. Additionally, the enrichment of *f*-rGO_DBSA_ with AgNWs led to a further reduction in the sheet resistance, even in the case of 10 wt % metal loading. The hybrid with 50 wt % silver content prepared using the in situ method exhibited the lowest sheet resistance, which was lower by 80% with respect to the *f*-rGO_DBSA_. In all cases, the AgNWs were well dispersed and anchored onto the surface of the graphene sheets, thus forming a highly interconnected 3D network that led to the enhanced surface conductivity we observed. Moreover, the incorporation of AgNWs was performed after following two synthetic strategies. A better electrical performance was revealed in the case of the “in situ” hybrids; this was attributed to the surface modification of AgNWs along with that of GO by DBSA, which led to their covalent attachment on the surface of the *f*-rGO_DBSA_ rather than to surface adsorption, as in the case of the “physical mixture” hybrids.

Additionally, the contribution of AgNWs to the improvement of the electrical properties of the graphene derivatives was further examined via the incorporation of preformed AgNWs in a hybrid of few-layered graphene with functionalized multi-walled carbon nanotubes. We observed that the sheet resistance of the FLG/MWNT-*f*-OH hybrid was also remarkably reduced after mixing with AgNWs. 

Given that the sheet resistance (R_s_) appeared to decrease when increasing the metal content, the *f*-rGO/AgNWs10% with a fairly low R_s_ was selected to be produced at a large scale for the preparation of a low-cost, water-based conductive ink. The as-prepared conductive inks were printed via gravure and found to be promising for applications in the field of printed electronics.

Overall, the graphene-based/AgNWs composites produced in the present work efficiently addressed the major technological barriers of state-of-the-art gravure printing inks, offering manifold advantages such as excellent water dispersibility without additives (e.g., surfactants); enhanced conductivity without post-treatment (e.g., sintering or annealing); and facile, cost-efficient, and scalable production. Further improvement of the hybrids’ dispersibility in the styrene–acrylate resin system is expected to optimize the printing quality.

## Figures and Tables

**Table 1:** EDS analysis of the FLG/MWNT-*f*-OH/AgNWs samples.

	Element % by Mass ^1^	
Samples	C	Ag
FLG/MWNT-*f*-OH/AgNWs_10_	94.42	5.58
FLG/MWNT-*f*-OH/AgNWs_30_	80.84	19.16
FLG/MWNT-*f*-OH/AgNWs_50_	74.79	25.21

^1^ Average values acquired from several regions.

## Data Availability

Data is contained within the article.
